# The complete chloroplast genome of *Scutellaria tsinyunensis* (Lamiaceae), an endemic species from China

**DOI:** 10.1080/23802359.2020.1781562

**Published:** 2020-06-24

**Authors:** Xiangning Liu, Youwei Zuo, Le Lin, Wenqiao Li, Qian Wang, Hongping Deng

**Affiliations:** a Chongqing Key Laboratory of Plant Resource Conservation and Germplasm Innovation, Institute of Resources Botany, School of Life Sciences, Southwest University, Chongqing, China; b Key Laboratory of Eco-Environments in Three Gorges Reservoir Region, School of Life Sciences, Southwest University, Chongqing, China

**Keywords:** *Scutellaria tsinyunensis*, chloroplast genome, phylogenetic analysis, Lamiaceae

## Abstract

*Scutellaria tsinyunensis* (Lamiaceae) is an endangered species endemic to Mt. Jinyun of Chongqing, China. In this study, the complete chloroplast (cp) genome of *S. tsinyunensis* was sequenced and characterized. The cp genome is 152,066 bp in length with a typical quadripartite structure, containing a pair of inverted repeats (IRs) of 25,223 bp separated by a large single-copy (LSC) region and a small single-copy (SSC) region of 84,096 bp and 17,524 bp, respectively. The whole cp genome contains 130 genes, including 86 protein-coding genes, 36 tRNA genes, and eight rRNA genes. The overall GC content of the circular genome is 38.4%, whereas the corresponding values in LSC, SSC, and IR regions are 36.4, 32.6, and 43.6%, respectively. The phylogenetic analysis based on the complete cp genomes of the Lamiaceae family indicated that *S. tsinyunensis* was closely related to *S. insignis*.

*Scutellaria* L. is a large, cosmopolitan genus of the Lamiaceae family, which includes nearly 360 species (Paton 1990). Most of them are widely distributed in Europe, North America, and East Asia (Paton 1990; Li et al. 2016). There are 98 species in China (Li and Ian 1994). *Scutellaria tsinyunensis* C. Y. Wu et S. Chow, a perennial herb, is exclusively distributed in Mt. Jinyun of Chongqing, China. Its poor sexual reproduction coupled with habitat degradation brought it near to extinction (Liu and Deng 2011; Zhang et al. 2011). Thus, it has been listed in the protected plants of Chongqing. To promote the conservation of this species, a comprehensive genetic resource is necessary to be conducted. In this study, we first reported and characterized the complete chloroplast (cp) genome of *S. tsinyunensis* based on Illumina paired-end sequencing data.

Fresh leave materials of *S. tsinyunensis* were collected from Beibei District, Chongqing, China (N29°50′18.82″, E106°23′31.92″). The voucher specimen (00198486) was deposited in the herbarium of Southwest University. Total genomic DNA was extracted using the modified CTAB method (Doyle and Doyle 1987). The whole-genome sequencing was conducted with 150 bp paired-end reads on the Illumina Hiseq X Ten platform at Biomarker Technologies (Beijing, China). The raw reads were filtered using Trimmomatic v0.32 (Bolger et al. 2014). The clean reads were then used to *de novo* assemble the complete chloroplast genome by SPAdes v3.12 (Bankevich et al. 2012). Annotation was performed with PGA (Qu et al. 2019) and Plann (Huang and Cronk 2015) with manual adjustments. The graphical map of the new cp genome was generated using OGDRAW v1.3.1 (Greiner et al. 2019).

The complete chloroplast genome of *S. tsinyunensis* (GenBank accession No. MT312247) is 152,066 bp in length with a typical quadripartite structure, containing a pair of inverted repeats (IRs) of 25,223 bp separated by a large single-copy (LSC) region and a small single-copy (SSC) region of 84,096 bp and 17,524 bp, respectively. The cp genome contains 130 genes, including 86 protein-coding genes, 36 tRNA genes, and eight rRNA genes. A total of 17 genes are duplicated in the IRs. In addition, among these annotated genes, 15 genes possess one intron, whereas two genes (*clpP* and *ycf3*) have two introns. The overall GC content of the circular genome is 38.4%, whereas the corresponding values in LSC, SSC, and IR regions are 36.4, 32.6, and 43.6%, respectively.

To identify the phylogenetic position of *S. tsinyunensis*, phylogenetic analysis was conducted. The maximum-likelihood (ML) tree with 1000 bootstrap replicates was reconstructed using RAxML (Stamatakis 2014) based on 36 complete cp genome sequences of Lamiaceae species (Figure 1). The result showed that *S. tsinyunensis* was closely related to *S. insignis*. The complete cp genome of *S. tsinyunensis* reported here provides a useful resource for the phylogenetic studies for Lamiaceae. Meanwhile, it also contributes to the conservation genetics of this species.

**Figure 1.**
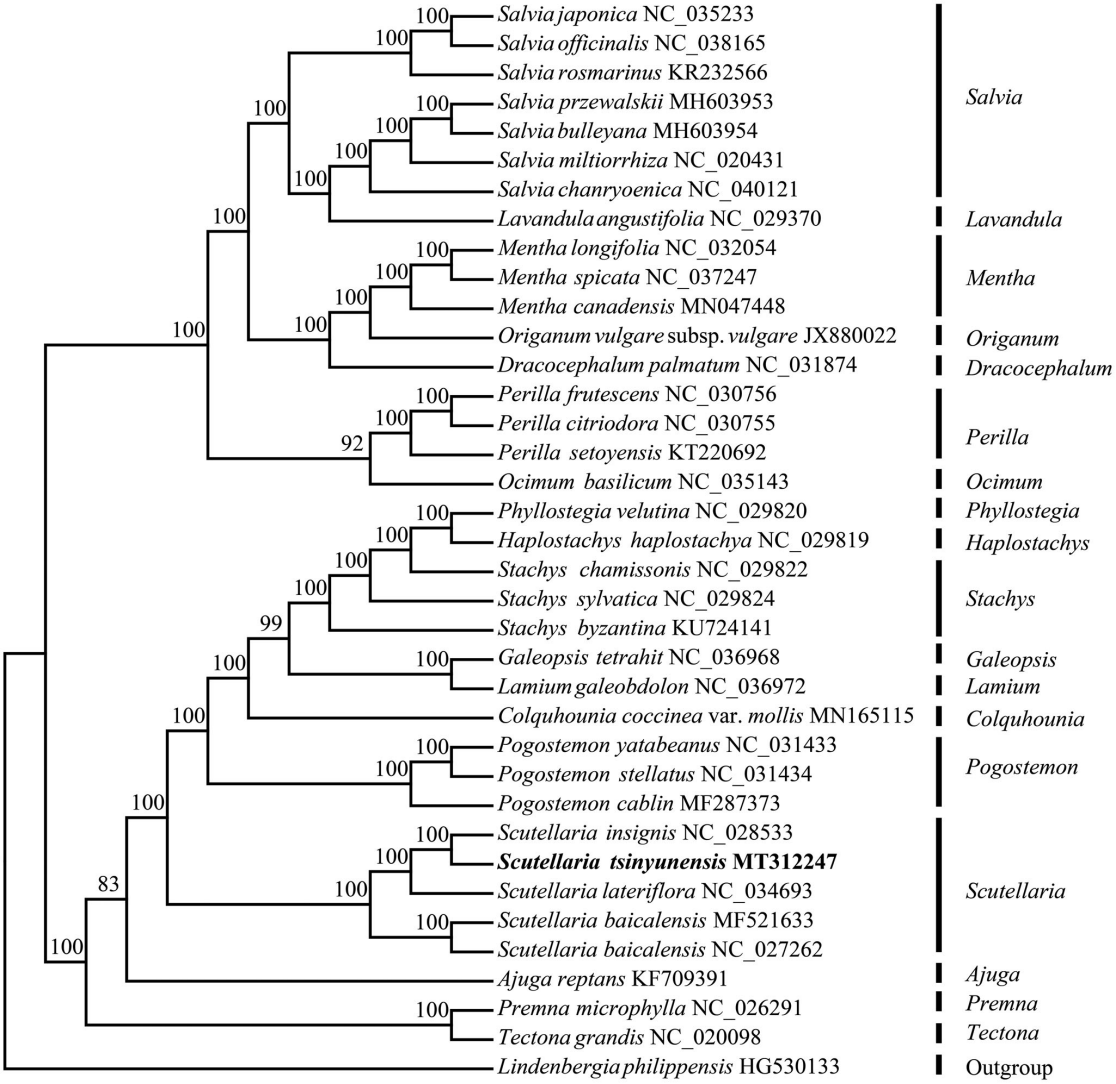
Maximum-likelihood (ML) tree of 35 species within the family Lamiaceae based on the complete chloroplast sequences using *Lindenbergia philippensis* as an outgroup. Numbers above the node indicate bootstrap values.

## Data Availability

The data that support the findings of this study are openly available in GenBank of NCBI at https://www.ncbi.nlm.nih.gov/, reference number [MT312247].
